# Attitude Correlated Frames Based Calibration Method for Star Sensors

**DOI:** 10.3390/s24010067

**Published:** 2023-12-22

**Authors:** Liheng Ma, Shenglong Xiao, Wenjun Tang, Xiao Luo, Su Zhang

**Affiliations:** 1 College of Ordnance Engineering, Naval University of Engineering, Wuhan 430033, China; 2 Guangzhou Bureau of the Department of Naval Equipment, Guangzhou 511464, China

**Keywords:** attitude-correlated frames, parameter calibration, star sensor, ACFCM, IAICM

## Abstract

Star sensors undergo laboratory calibration before they leave the factory. In addition, recalibration is necessary after they experience vibration, deformation, etc. Using the analysis of attitude-dependent and attitude-independent interstar angular invariance calibration methods (IAICMs) as a reference, an attitude-correlated frame-based calibration method (ACFCM) is proposed in this work, which combines the advantages of both methods. Using outdoor star observations, the ACFCM correlates star image frames obtained at different times via a strapdown gyro unit. As a result, the number of efficient star images for calibration increases rapidly and the distribution of star images becomes much more uniform, thus improving the calibration accuracy of the star sensor. A simulation and experimental tests were designed and carried out. Both the simulation and experimental results verify the feasibility of the proposed ACFCM method. Furthermore, by comparing our method with the IAICMs, the repeatability and reliability of the principal point obtained from the calibration with the ACFCM method proposed in this work were significantly improved.

## 1. Introduction

Star sensors with the highest absolute attitude measurement accuracy establish the attitude relationship between the body coordinate system of the star sensor and the celestial sphere inertial coordinate system using a procedure that involves imaging the light of the star, star centroiding, and star identification. The attitude accuracy of the star sensor is able to reach the subarcsecond level. Thus, it has been used in precise attitude measurements and attitude calibrations in deep-space exploration, satellites, ballistic missiles, aircraft, and ships [1,2]. The accurate calibration of the star sensor is a prerequisite for its operation [3]; otherwise, there will be computational errors in its observation vectors, which will affect the accuracy of star centroiding, star identification, and the subsequent attitude determination. These errors will lead to a decrease in performance or even the sensor being unable to work normally [4]. Therefore, star sensors are generally subjected to precision indoor calibration before leaving the factory, and they need to be calibrated again, for example, an on-orbit calibration [5], after being disturbed by external factors such as launch vibration and temperature deformation.

Star sensor calibration methods can be divided into two categories [6]: the method of undetermined coefficients, in which the image sensitivity plane is polynomial fitted, and the internal parameter calibration method, in which the parameters of the star sensor, including the principal point, focal length, and distortion of the optical system, are estimated. Certain pre-products of star sensors [6,7] use the undetermined coefficients method, due to the FOVs of these products being relatively small; however, the major limitation of this method is the small size of the curvature it constructs. Therefore, it is difficult to obtain an accurate surface approximation in the full FOV range for big FOVs. As a result, the internal parameter calibration method is utilized in star sensors with big FOVs. In this paper, as described below, the calibration method is specified for internal parameter calibration as most of the star sensors at present have a big FOV.

Depending on the calibration location and observation objects, star sensor internal parameter calibration methods can be divided into laboratory calibration and outdoor star observation calibration. Laboratory calibration relies on a single-star or multi-star simulator to simulate the starlight. A multi-star simulator can directly simulate different celestial sphere regions under different times and viewing positions. Therefore, the calibration process is easy to carry out. The performance of a dynamic multi-star simulator is better. However, simulators are expensive. The simplest single-star simulator can be realized using a collimator [8], which is generally required to have a focal length approximately 10 times that of the star sensor being calibrated [9] in order to ensure the parallelism of the incident light. The optical axes of the collimator and the star sensor are adjusted, and when the two are parallel, the position of the imaging point denotes the principal point. However, the adjustment is very difficult. By rotating the star sensor 360 degrees around the optical axis, the imaging points form an approximate circle, and the center of the circle denotes the principal point of the star sensor [6]. Moreover, the calibration of the focal length can be obtained from the relationship between the object and its image, such as the object size relationship of the cross-bar in the collimator. The distortion calibration depends on multiple point images on the target surface of the image sensor and requires the selection of an appropriate distortion model [10]. The principal point and focal length can also be calibrated using the multi-point imaging relationship. The imaging position of the star spot on the image sensor needs to be controlled using the rotation of a precision 2D rotation table [9]; thus, a precision rotation table is also necessary equipment for calibration. This type of calibration is known as attitude-dependent calibration.

The laboratory calibration method with the single-star simulator and precision 2D rotation table has become the domestic industry standard [9] in China. As an alternative option to the expensive precision rotation table, a 2D adjustable plane mirror is installed between the single-star simulator and the star sensor to control the direction of the incident light in [11]. The coupling between the parameters of the star sensor makes the calibration method for individual parameters less reliable. A four-step laboratory calibration method is proposed in [12], which can achieve global optimization of all parameters.

Outdoor star observation calibration works by directly imaging starlight; thus, it does not rely on equipment such as single- or multiple-star simulators or precision rotation tables. Therefore, it is regarded as attitude-independent. The principle of interstar angular invariance is adopted in outdoor star observation calibration [13,14,15]. The interstar angular invariance calibration method (IAICM) is easy to carry out, but it is sensitive to noise interference, and the accuracy of the calibration is reduced. Depending on the calibration location, the IAICM can be divided into on-orbit calibration methods [13,15,16] and ground-star observation calibration methods [11,17]. On-orbit calibration methods use full auto-calibration and there is no external intervention [5]. The iteration calibration method, which considers the weight of different stars, is proposed in [18]. In addition, an angular distance subtraction on-orbit calibration method is presented in [19].

The attitude-dependent calibration method requires a bigger calibration setup, such as a high-precision rotation table. Moreover, for the attitude-independent method, namely the IAICM, the principal point calibration result is not stable and the number of efficient stars for calibration is not sufficient. After analyzing these calibration methods, an attitude-correlated frame-based calibration method (ACFCM) is proposed. The ACFCM combines the advantages of the IAICM and the laboratory attitude-dependent calibration method. It does not require an expensive calibration setup, but it achieves the attitude measurement using the strapdown gyro unit. Using outdoor star observation, the ACFCM correlates star image frames obtained at different times through the strapdown gyro unit. Meanwhile, the correlation time of the ACFCM is determined by the number of attitude maneuvers, the duration time of each attitude status, and the gyro errors. For a 50-type laser gyro unit (LGU) used in this work, the gyro errors can be ignored for about 9 min. As a result, the number of efficient stars for calibration increases rapidly and the distribution of star images becomes much more uniform, which is beneficial for the calibration; thus, the calibration accuracy of the principal point increases. Our method is especially suitable for star sensors with a strapdown inertial navigation system (SINS) since the ACFMC requires a gyro unit.

The remainder of this work is organized as follows: In Section 2, with the introduction of the star sensor measurement model, the attitude-dependent and attitude-independent calibration methods are summarized and analyzed. Then, the proposed calibration method based on the ACF approach is presented in Section 3. The simulation and experiment are described in Section 4, which verify the proposed calibration method. Finally, conclusions are drawn in Section 5.

## 2. Previous Work

### 2.1. Measurement Model of the Star Sensor

**Figure 1:**
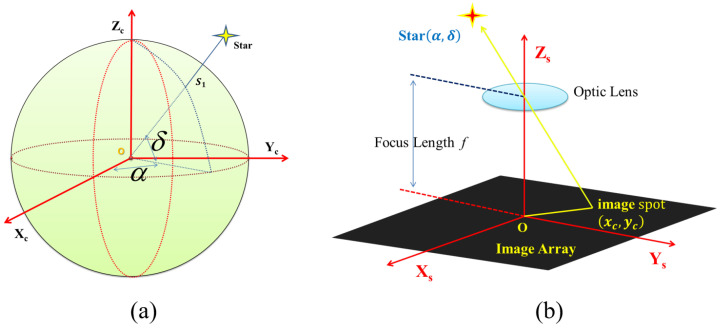
Schematic diagram of an attitude measurement for the star sensor: (**a**) inertial celestial sphere coordinates and the coordinate of a star and (**b**) star sensor coordinates and the projection of a star.

The star sensor establishes the attitude relationship between the body coordinate system of the star sensor (s-coordinates) and the inertial celestial sphere coordinate system (i-coordinates). Figure 1 shows the schematic diagram of attitude measurement for the star sensor. A star with right ascension and declination (α,δ) in the i-coordinates is imaged on the target surface of the image sensor through its optical system to form the star image. After star image processing and star centroiding, and with the use of the parameters of the star sensor, the observation vector of a star can be obtained:

(1) $$W=\frac{1}{\sqrt{{\left(x_{c}-x_{0}\right)}^{2}+{\left(y_{c}-y_{0}\right)}^{2}+f^{2}}}\left(\begin{array}{c} -(x_{c}-x_{0})\\ -(y_{c}-y_{0})\\ f \end{array}\right),$$

where $\left(x_{0},y_{0}\right)$ is the principal point of the star sensor, *f* is the equivalent focal length of the lens group, and $\left(x_{c},y_{c}\right)$ is the centroids of a star image in the s-coordinates. Distortions will contribute to the error of the star centroids and should be compensated for with an appropriate distortion model. Moreover, the corresponding reference vector of the star is

(2) $$V=\left(\begin{array}{c} cos\alpha \cdot cos\delta \\ sin\alpha \cdot cos\delta \\ sin\delta \end{array}\right).$$

The reference vectors in the i-coordinates and the observation vectors in the s-coordinates are linked by the attitude matrix $C_{s}^{i}$ in Equation (3):

(3) $$V=C_{s}^{i}\cdot W+E,$$

where E is the measurement error of the star sensor, and the attitude matrix $C_{s}^{i}$ represents the attitude transform from the s-coordinates to the i-coordinates.

The principal point, the focal length, and the coefficients in the distortion model are the internal parameters of the star sensor that need to be calibrated.

### 2.2. Attitude-Dependent Calibration Method

The basic idea of the attitude-dependent calibration method is to compare the measured observation vectors and the computed vectors, which is achieved using the attitude equation, i.e., Equation (3). The attitude-dependent calibration method is regarded as the Chinese national industry standard “Calibration and Accuracy Test Method for Star Sensors” [9], and, as an example, it is described below.

The calibration is performed in the laboratory by combining a single-star simulator and a rotation table. The star sensor is fixed on a two-dimensional rotation table, which can rotate around its *x* and *y* axes, and the single-star simulator is placed in front of the star sensor to simulate the incidence of starlight, with the angle between the incidence direction and the optical axis *z* of the star sensor being (β,γ). Consider the three-axis mounting error angle between the star sensor and the rotation table, i.e., the external parameter, which is set as $\left(\phi_{1},\phi_{2},\phi_{3}\right)$, corresponding to the *x*, *y*, and *z* axes, respectively. The rotation table is rotated at certain intervals so that the star images of the single-star simulator can form a point map covering the entire image plane of the star sensor, and the center-of-mass method is used to obtain the centroids of the star images. As a result, the imaging model of the incident light, i.e., the calibration model, can be established:

(4) $$\left(\begin{array}{c} f_{1}\\ f_{2}\\ f_{3} \end{array}\right)=R_{RS}\left(\phi_{1},\phi_{2},\phi_{3}\right)R_{rot}\left(\theta_{1},\theta_{2}\right)\left(\begin{array}{c} cos\beta \cdot cos\gamma \\ sin\beta \cdot cos\gamma \\ sin\gamma \end{array}\right),$$

where $\left(\theta_{1},\theta_{2}\right)$ are the angles rotated by the rotation table during the calibration process, which are recorded simultaneously. Then, the centroids of the star images $\left(x_{r},y_{r}\right)$ can be presented by the parameters in Equation (4) as

(5) $$\left\{\begin{array}{c} x_{r}=x_{0}+f\frac{f_{1}}{f_{3}}+dx\\ y_{r}=y_{0}+f\frac{f_{2}}{f_{3}}+dy \end{array}\right.,$$

where (dx,dy) is the distortion term. There are five unknowns, i.e., $\left(\phi_{1},\phi_{2},\phi_{3},\beta ,\gamma \right)$, in Equation (5), plus three internal parameters, i.e., $\left(x_{0},y_{0},f\right)$. Thus, there is a total of eight unknowns to be solved. If the distortion term (dx,dy) is not taken into account, then at least four star data images are needed to establish the above parameters. Further, once the distortion is taken into consideration, the observations need to be increased correspondingly according to the number of coefficients in the distortion model. In the actual calibration process, the rotation table is rotated so that the star images are regularly spread over the whole FOV. Generally, star images in the same position are imaged several times, and the average value of their centroids is adopted as the final measurement in order to reduce noise interference and to improve the accuracy of the calibration result.

The theoretically computed observed values $\left(x_{r},y_{r}\right)$ can be obtained using Equation (5). They are compared with the measured values $\left(x_{m},y_{m}\right)$, as shown in Equation (6), in which the higher-order terms have been ignored and only linear terms are maintained:

(6) $$\left\{\begin{array}{c} x_{r}-x_{m}=G_{x}\cdot ∆p\\ y_{r}-y_{m}=G_{y}\cdot ∆p \end{array}\right.,$$

where $∆p={\left[∆x_{0},∆y_{0},∆f,∆\alpha ,∆\beta ,∆\phi_{1},∆\phi_{2},∆\phi_{3}\right]}^{T}$, $G_{x}$, and $G_{y}$ are the sensitive matrices; here, the distortion terms are omitted for simplicity. The sensitive matrix is

(7) $$\left\{\begin{array}{c} G_{x}=\left(\begin{array}{c} \frac{\partial f_{x}}{\partial x_{0}},\frac{\partial f_{x}}{\partial y_{0}},\frac{\partial f_{x}}{\partial f},\frac{\partial f_{x}}{\partial \alpha },\frac{\partial f_{x}}{\partial \beta },\frac{\partial f_{x}}{\partial \phi_{1}},\frac{\partial f_{x}}{\partial \phi_{2}},\frac{\partial f_{x}}{\partial \phi_{3}} \end{array}\right)\\ G_{y}=\left(\begin{array}{c} \frac{\partial f_{y}}{\partial x_{0}},\frac{\partial f_{y}}{\partial y_{0}},\frac{\partial f_{y}}{\partial f},\frac{\partial f_{y}}{\partial \alpha },\frac{\partial f_{y}}{\partial \beta },\frac{\partial f_{y}}{\partial \phi_{1}},\frac{\partial f_{y}}{\partial \phi_{2}},\frac{\partial f_{y}}{\partial \phi_{3}}, \end{array}\right) \end{array}\right.,$$

where $f_{x}$ and $f_{y}$ are functions defined by the terms on the right-hand side of Equation (5) with respect to *x* and *y*.

Given the initial value of the internal parameters, a set of ∆p values can be obtained by solving Equation (6). An iteration process is employed to update the value of ∆p and a high-accuracy calibration result is obtained. According to the relevant literature [9], the root-mean-square (RMS) values of the calibration residuals of the theoretically computed star image centroids, and the measured values are x<0.0412 pixels and y<0.0434 pixels, respectively. However, an obvious shortcoming of the attitude-dependent calibration method is that the internal and external parameters are easily coupled; thus, a decoupled calibration method was also studied [20].

### 2.3. Attitude-Independent Calibration Method

Attitude-independent calibration methods specifically refer to a regular interstar angle invariable calibration method (IAICM) [13]. These are based on the principle of constant interstellar angular distance and are commonly used for the on-orbit calibration of star sensors.

According to the attitude equation, i.e., Equation (3), assuming that the observation vectors corresponding to star images i and j on the image plane are $W_{i}$ and $W_{j}$, and the reference vectors are $V_{i}$ and $V_{j}$, respectively, the attitude matrix $C_{s}^{i}$ has the quality of orthogonality; thus, the following relationship is satisfied:

(8) $$W_{i}^{T}\cdot W_{j}={\left(C_{s}^{i}\cdot V_{i}\right)}^{T}\cdot C_{s}^{i}\cdot V_{j}=V_{i}^{T}\cdot V_{j}.$$

Equation (8) is the principle of interstar angular distance invariance. In contrast to the attitude-dependent calibration method, there no longer exists attitude information, which makes the calibration process relatively simple and especially suitable for on-orbit calibration.

The specific form of the observation vector Equation (1) and reference vector Equation (2) are substituted into Equation (8) as follows:

(9) $$V_{i}^{T}\cdot V_{j}=\frac{D_{ij}}{N_{i}N_{j}}=G_{ij}\left(x_{0},y_{0},f\right),$$

where

(10) $$D_{ij}=\left(x_{i}-x_{0}\right)\left(x_{j}-x_{0}\right)+\left(y_{i}-y_{0}\right)\left(y_{j}-y_{0}\right)+f^{2}$$

(11) $$N_{i}=\sqrt{{\left(x_{i}-x_{0}\right)}^{2}+{\left(y_{i}-y_{0}\right)}^{2}+f^{2}}$$

(12) $$N_{j}=\sqrt{{\left(x_{j}-x_{0}\right)}^{2}+{\left(y_{j}-y_{0}\right)}^{2}+f^{2}}.$$

In addition, let $x_{0}={\tilde{x}}_{0}+∆x_{0}$, $y_{0}={\tilde{y}}_{0}+∆y_{0}$ and $f=\tilde{f}+∆f$, ${\tilde{x}}_{0}$, ${\tilde{y}}_{0}$, and $\tilde{f}$ be the estimates of the principal points and focal lengths, respectively. Thus, $∆x_{0}$, $∆y_{0}$, and ∆f are the errors between the estimates and the true values $x_{0}$, $y_{0}$, and *f*. By linearly expanding $G_{ij}\left(x_{0},y_{0},f\right)$, we can obtain:

(13) $$\begin{array}{cc} G_{ij}\left(x_{0},y_{0},f\right) & =G_{ij}\left({\tilde{x}}_{0},{\tilde{y}}_{0},\tilde{f}\right)\\ \; & +\left(\frac{\partial G_{ij}}{\partial x_{0}}\;\frac{\partial G_{ij}}{\partial y_{0}}\;\frac{\partial G_{ij}}{\partial f}\right)\left(\begin{array}{c} ∆x_{0}\\ ∆y_{0}\\ f \end{array}\right) \end{array}.$$

In addition, let

(14) $$\begin{array}{cc} R_{ij} & =V_{i}^{T}V_{j}-G_{ij}\left({\tilde{x}}_{0},{\tilde{y}}_{0},\tilde{f}\right)\\ \; & =\left(\frac{\partial G_{ij}}{\partial x_{0}}\;\frac{\partial G_{ij}}{\partial y_{0}}\;\frac{\partial G_{ij}}{\partial f}\right)\left(\begin{array}{c} ∆x_{0}\\ ∆y_{0}\\ f \end{array}\right) \end{array}.$$

Considering the interstar distance between multiple stars, the above equation can be expanded as

(15) $$\left(\begin{array}{c} R_{12}\\ R_{13}\\ :\\ R_{ij} \end{array}\right)=\left(\begin{array}{ccc} \frac{\partial G_{12}}{\partial x_{0}} & \frac{\partial G_{12}}{\partial y_{0}} & \frac{\partial G_{12}}{\partial f}\\ \frac{\partial G_{13}}{\partial x_{0}} & \frac{\partial G_{13}}{\partial y_{0}} & \frac{\partial G_{13}}{\partial f}\\ : & : & :\\ \frac{\partial G_{ij}}{\partial x_{0}} & \frac{\partial G_{ij}}{\partial y_{0}} & \frac{\partial G_{ij}}{\partial f} \end{array}\right)\left(\begin{array}{c} ∆x_{0}\\ ∆y_{0}\\ f \end{array}\right).$$

Furthermore, simplify Equation (15) as

(16) R=H·∆Z.

Here, the matrix H is the sensitive matrix, and the values of $∆Z={\left(∆x_{0},∆y_{0},∆f\right)}^{T}$ can be estimated using the least square method. Then, the estimated ∆Z is utilized to compensate for ${\tilde{x}}_{0},{\tilde{y}}_{0},\tilde{f}$:

(17) $${\left({\tilde{x}}_{0},{\tilde{y}}_{0},\tilde{f}\right)}_{i+1}={\left({\tilde{x}}_{0},{\tilde{y}}_{0},\tilde{f}\right)}_{i}+\left(∆x_{0},∆y_{0},∆f\right).$$

The subscript *i* and i+1 are the index of iteration. With the updated values of ${\tilde{x}}_{0},{\tilde{y}}_{0},\tilde{f}$, the iteration of solving Equation (16) continues until the calibration result is accurate enough or the number of iteration reaches the upper limit that is set in advance.

### 2.4. Analysis of the Sensitive Matrix

Through the analysis of the sensitive matrix of the calibration methods presented above, we can take a close look at their performance. First, taking the elements $\frac{\partial G_{ij}}{\partial x_{0}}$, $\frac{\partial G_{ij}}{\partial y_{0}}$, and $\frac{\partial G_{ij}}{\partial f}$ from H in the IAICM as an example, let $\left(x_{0},y_{0}\right)$ be the origin of the s-coordinates, and only consider the *x* component for simplicity because of the symmetry of *x* and *y*. Then, the elements can be written as

(18) $$\left\{\begin{array}{c} \frac{\partial G_{ij}}{\partial x_{0}}=\frac{N_{i}^{{}^{\prime }}N_{j}^{{}^{\prime }}\cdot \left(-x_{i}-x_{j}\right)+D_{ij}^{{}^{\prime }}\left(x_{i}N_{j}^{{}^{\prime }}/N_{i}^{{}^{\prime }}+x_{j}N_{i}^{{}^{\prime }}/N_{j}^{{}^{\prime }}\right)}{{\left(N_{i}^{{}^{\prime }}N_{j}^{{}^{\prime }}\right)}^{2}}\\ \frac{\partial G_{ij}}{\partial f}=\frac{N_{i}^{{}^{\prime }}N_{j}^{{}^{\prime }}\cdot 2f-\left(D_{ij}^{{}^{\prime }}fN_{j}^{{}^{\prime }}/N_{i}^{{}^{\prime }}+N_{i}^{{}^{\prime }}/N_{j}^{{}^{\prime }}\right)}{{\left(N_{i}^{{}^{\prime }}N_{j}^{{}^{\prime }}\right)}^{2}} \end{array}\right.$$

and

(19) $$\left\{\begin{array}{c} N_{i}^{{}^{\prime }}=\sqrt{x_{i}^{2}+f^{2}}\\ N_{j}^{{}^{\prime }}=\sqrt{x_{j}^{2}+f^{2}}\\ D_{ij}^{{}^{\prime }}=x_{i}x_{j}+f^{2} \end{array}\right..$$

Therefore, the calibration error is

(20) $$\delta R_{ij}=\sqrt{2\cdot {\left(\frac{\partial G_{ij}}{\partial x_{0}}∆x_{0}\right)}^{2}+{\left(\frac{\partial G_{ij}}{\partial f}∆f\right)}^{2}}.$$

**Figure 2:**
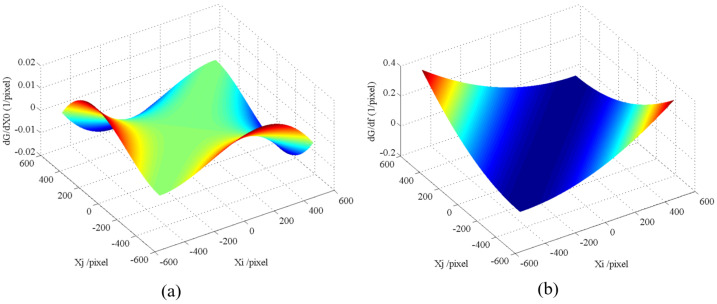
Relationship between the star $\left(x_{i},x_{j}\right)$ and the sensitive matrix term of the (**a**) principal point and (**b**) focus length. Different colors represent different values of the partial differential.

With *f* = 25.6 mm, −3.3 mm $<x_{i},x_{j}<$ 3.3 mm, and a pixel size *d* = 6.45 μm, the relationship between $\partial G_{ij}/\partial x_{0}$, $\partial G_{ij}/\partial f$ and $x_{i}$, $x_{j}$ can be obtained, as shown in Figure 2.

It can be seen from Figure 2 that the values of $\partial G_{ij}/\partial x_{0}$ are ten times smaller than that of $\partial G_{ij}/\partial f$. When a variable is multiplied to $\partial G_{ij}/\partial x_{0}$ and $\partial G_{ij}/\partial f$ with the same value, the change in the product for $\partial G_{ij}/\partial f$ is much bigger. Therefore, the IAICM error is very insensitive to the principal point since the values of the sensitive matrix are very small, as shown in Figure 2a. This means that even if the error of the principal point $∆x_{0}$ is a larger value, the corresponding term of the sensitivity matrix will not be sensitive to the change, which increases the degree of uncertainty of the principal point $∆x_{0}$ solution. Regarding the focus length, the values of the sensitivity matrix are large, as shown in Figure 2b. Therefore, it will be sensitive to the change in the focus length, and the calibration error of the focal length will be stable. The study in [21] obtained the same conclusion.

For the sensitive matrices in the attitude-dependent calibration method, i.e., $G_{x}$ and $G_{y}$ in Equation (6), the attitude-dependent parameters in Equation (7), $\partial f/\partial \alpha ,\partial f/\partial \beta ,\partial f/\partial \phi_{1},$$\partial f/\partial \phi_{2},\partial f/\partial \phi_{3}$, are ignored in order to remain consistent with those in the H matrix and be able to better compare them.

(21) $$\left\{\begin{array}{c} G_{x}=\left[1,0,f_{1}/f_{3}\right]\\ G_{y}=\left[0,1,f_{2}/f_{3}\right] \end{array}\right.$$

According to Equation (4), since the angle of incidence β is generally large and remains around 90 degrees, $\phi_{1},\phi_{2},\phi_{3}\approx 0$, i.e., the matrix $R_{RS}\left(\phi_{1},\phi_{2},\phi_{3}\right)\approx I_{3\times 3}$. Therefore, $f_{1},f_{2}<f_{3}$, and the values in the sensitive matrix related to the principal point are larger than those related to the focal length. Therefore, the sensitive matrices $G_{x}$ and $G_{y}$ in the attitude-dependent calibration method are more sensitive to the principal point. As a result, the principal point calibration result is much more reliable compared to that of the IAICM.

## 3. Attitude-Correlated Frame-Based Calibration Method

The attitude-dependent calibration method achieves highly accurate calibration results with a star simulator and rotation table in the laboratory. Through direct star observation, the IAICM attitude-independent calibration method no longer depends on high-precision calibration equipment. However, the principal point calibration result produced by the IAICM is inaccurate. The ACFCM proposed in this work possesses the advantages of both calibration methods. Using our method, the calibration process is completed with direct star observations like the IAICM; therefore, there is no need for precision calibration equipment. Different star image frames are correlated; thus, the number of star images for calibration increases rapidly, and the distribution of the star images in the correlated frame is distributed uniformly as in the attitude-dependent calibration method. As a result, the principal point calibration accuracy with the ACFCM is more stable than with the IAICM.

### 3.1. Principle of the ACF Approach

**Figure 3:**
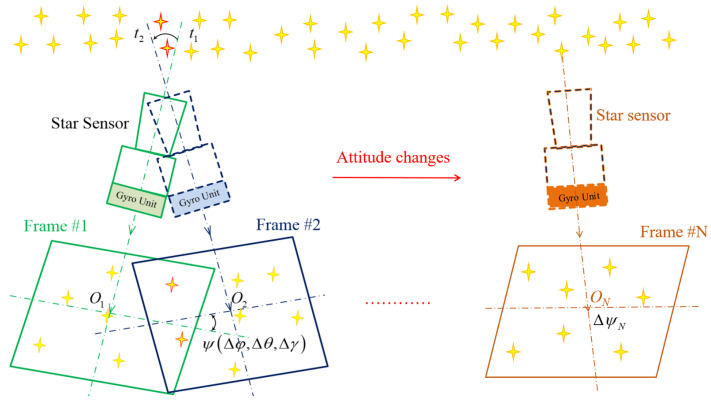
Schematic diagram of the attitude-correlated frames approach.

The attitude-correlated frames (ACF) [2,22,23] method was initially proposed to improve the dynamic performance of star sensors. The ACF method addresses the limitations of a single-star image frame and expands to a sequence of adjacent star image frames. A big star image frame, including a much more efficient star, is generated and can be used for calibration, as illustrated in Figure 3. In addition, the attitude relationship between different star image frames can be obtained using the strapdown gyro unit.

The cross-boresight (corresponding to the *x* and *y* axes) noise equivalent angle (NEA) error $E_{ss}$ can be used to evaluate the accuracy of the star sensor, and it is calculated as follows [1]:

(22) $$E_{ss}=\frac{FOV_{1D}}{n_{pixel}}\frac{E_{centroid}}{\sqrt{{\bar{n}}_{star}}},$$

where $FOV_{1D}$, given in degrees, is the field of view of the star sensor in one dimension, $n_{pixel}$ is the one-dimensional resolution of the image sensor in the star sensor in pixels, $E_{centroid}$ is the average centroiding accuracy in units of pixels, and ${\bar{n}}_{star}$ is the average number of detected stars per frame.

A sequence of star image frames can be correlated with the ACF approach. After motion compensation, star centroiding, and star identification, the observed vector sequence and the reference vector sequence of the star image frames $V^{j},W^{j},\left(j=1,2\cdots ,N\right),j$ are obtained, respectively (j is the star image frame index number in the frame sequence). The attitude transforms of the star image frames, $G_{k}^{N}\left(k=1,2,\cdots ,N-1\right)$, are calculated using the gyro unit, and the attitude equation of the *N*th frame with the ACF approach can be given by

(23) $$\left\{\begin{array}{c} \left[V^{1},V^{2},\cdots ,V^{N}\right]=C_{sN}^{i}\left[G_{1}^{N}W^{1},G_{2}^{N}W^{2},\cdots ,W^{N}\right]\\ +[E^{1},E^{2},\cdots ,E^{N}]\\ G_{k}^{N}=\prod_{l=k}^{l=N-1}G_{l}^{l+k},k=1,2,\cdots ,N-1 \end{array}\right.,$$

where $E^{1},E^{2},\cdots ,E^{N}$ are the measuring errors of the star sensor itself. The errors in $G_{k}^{N}$(k=1,2,⋯,N−1) caused by the error of the gyro unit are discussed in [22]. The accumulated errors during the correlation time for a 50-type LGU can be ignored when the correlation time is not too long. According to [22], the correlation time is 500 s for the 50-type LGU with an accuracy expectation of 5 arcseconds for the star sensor. Therefore, a much larger star image frame of $N\cdot {\bar{n}}_{star}$ stars is generated with the ACF approach, and Equation (22) can be rewritten as

(24) $$E_{ss}=\frac{FOV_{1D}}{n_{pixel}}\frac{E_{centroid}}{\sqrt{{\bar{n}}_{star}\cdot N}},$$

where N is the number of correlated star image frames used in the ACF method.

As a result, the attitude error in Equation (24) will be reduced by a factor of $\sqrt{N}$. Thus, in order to reduce the attitude error further, we can increase the number of correlated frames. Moreover, the number of star images in the correlated frame will also increase, which is beneficial for calibration.

### 3.2. The Proposed Calibration Method

According to the attitude-dependent calibration model in Equation (5), the following mapping relations are obtained for the *j*th star image on the *k*th image frame of the star sensor:

(25) $$\left\{\begin{array}{c} x_{r}^{kj}=x_{0}+f\frac{f_{1}^{kj}}{f_{3}^{kj}}+dx\\ y_{r}^{kj}=y_{0}+f\frac{f_{2}^{kj}}{f_{3}^{kj}}+dy \end{array}\right..$$

Here, $\left(x_{0},y_{0}\right)$ remains the principal location, and dx and dy are the distortions and can be described by the distortion model:

(26) $$\left\{\begin{array}{c} dx=\bar{x}\left(q_{1}r^{2}+q_{2}r^{4}\right)+p_{1}\left(r^{2}+2{\bar{x}}^{2}\right)+2p_{2}xy\\ dy=\bar{y}\left(q_{1}r^{2}+q_{2}r^{4}\right)+p_{2}\left(r^{2}+2{\bar{y}}^{2}\right)+2p_{1}xy \end{array}\right.,$$

where $\bar{x}=x_{r}^{kj}-x_{0}$, $\bar{y}=y_{r}^{kj}-y_{0}$, $r^{2}={\bar{x}}^{2}+{\bar{y}}^{2}$, $q_{1}$ and $q_{2}$ are radial distortion coefficients, and $p_{1}$ and $p_{2}$ are tangential distortion coefficients. In addition, ${\left[f_{1}^{kj},f_{2}^{kj},f_{3}^{kj}\right]}^{T}$ in Equation (25) is the projection vector of the *j*th star reference vector on the *k*th image frame in the star catalog:

(27) $${\left[f_{1}^{kj},f_{2}^{kj},f_{3}^{kj}\right]}^{T}=V^{kj}=C_{i}^{k}W^{kj},$$

where the attitude matrix $C_{i}^{k}$ denotes the attitude transform matrix from the i-coordinates to the s-coordinates for the *k*th star image frame.

The attitude matrix is obtained by recording the rotation angles of the precision rotation table for the laboratory attitude-dependent calibration method described in Section 2.2. Moreover, the ACFCM adopts the outdoor star observation method and no longer depends on precision calibration equipment, while the high-precision attitude information is calculated by the ACF approach. According to Equation (23), the attitude matrix $C_{i}^{k}$ can, therefore, be optimized by the ACF method:

(28) $$\begin{array}{cc} \left[V^{1},V^{2},\cdots ,V^{k}\right] & ={\tilde{C}}_{k}^{i}\left[G_{1}^{k}W^{1},G_{2}^{k}W^{2},\cdots ,W^{k}\right]\\ \; & +[E^{1},E^{2},\cdots ,E^{k}], \end{array}$$

where ${\tilde{C}}_{k}^{i}$ is an estimate of the attitude matrix $C_{k}^{i}$ and ${\tilde{C}}_{i}^{k}={\tilde{C}}_{k}^{i}$. In addition, the correlation matrix $G_{n}^{k}$ can be specifically expressed as

(29) $$G_{n}^{k}=C_{g}^{s}C_{n}^{k}{\left(C_{g}^{s}\right)}^{T},$$

where $C_{g}^{s}\left(\phi_{1},\phi_{2},\phi_{3}\right)$ is the mounting matrix between the star sensor and the gyro unit, and $C_{n}^{k}$ is the attitude change between the *k*th and *n*th frames calculated using the gyro unit.

Unlike the iteration estimation procedure in Section 2.2 and Section 2.3, here, the parameters to be calibrated are estimated in a non-linear optimization manner by solving the objective function. Let the measured centroids of the *j*th star image on the *k*th image frame be $\left(x_{m}^{kj},y_{m}^{kj}\right)$, and then the objective function can be set up between the measured star centroids and the projected values $\left(x_{r}^{kj},y_{r}^{kj}\right)$ obtained by the model in Equation (27). Then, for multiple frames, we have:

(30) $$\begin{array}{c} \varepsilon (x_{0},y_{0},f,q_{1},q_{2},p_{1},p_{2},\phi_{1},\phi_{2},\phi_{3})\\ =min\left\{\sum_{k=1}^{N}\sum_{j=1}^{n}\left[{\left(x_{m}^{kj}-x_{r}^{kj}\right)}^{2}+{\left(y_{m}^{kj}-y_{r}^{kj}\right)}^{2}\right]\right\} \end{array},$$

where $\left(x_{0},y_{0},f,q_{1},q_{2},p_{1},p_{2},\phi_{1},\phi_{2},\phi_{3}\right)$ are the parameters to be calibrated, and the above objective function can be solved using an optimized algorithm such as the Levenberg–Marquardt algorithm. When the angles in the mounting matrix are measured in advance, the parameters to be calibrated are $\left(x_{0},y_{0},f,q_{1},q_{2},p_{1},p_{2}\right)$.

The specific calibration step can be concluded as follows. First, give the initial values of the parameters to be calibrated and input the information of the star image frame; then, the attitude matrix $C_{i}^{k}$ is obtained by solving Equation (28). Second, the objective function Equation (30) is solved using the Levenberg–Marquardt algorithm to obtain a set of calibration results of the parameters. Third, the attitude matrix $C_{i}^{k}$ is recalculated with the parameters obtained from the solution in the second step using the ACF approach. Fourth, repeat the second and third steps until the error ε changes before and after the calibration, which is less than the previously set threshold, and, finally, output the calibration results of the parameters.

## 4. Experiment and Results

### 4.1. Simulation

**Figure 4:**
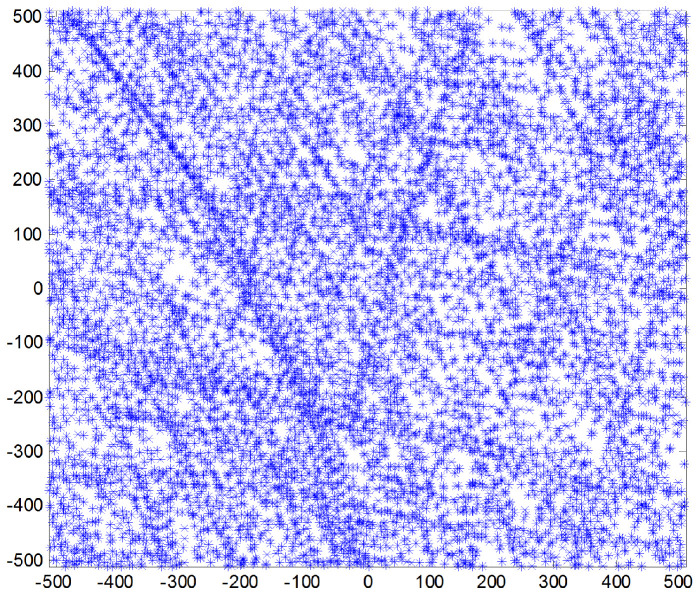
Star distribution with the ACF approach.

The initial attitude and parameters of the star sensor were set and are listed in Table 1. The gyro unit was rotated around its three axes at an angular rate of 1${}^{\circ }$/s to generate the track information, with which the corresponding star image frames were produced. The gyro bias was 0.01${}^{\circ }$/h and the random walk noise was set as 0.003${}^{\circ }/\sqrt{h}$. The star image frames at different times were correlated and a much bigger frame was obtained, as shown in Figure 4. The star image number on this new frame increased sharply and was distributed more uniformly.

Different levels of Gaussian white noise were applied to the star sensor centroids, with a noise mean value of zero and a standard deviation of 0 pixels (Group 1), 0.1 pixels (Group 2), and 0.2312 pixels (Group 3). Star sensor calibration was carried out using the IAICM method and the ACFCM method proposed in this work. For each noise level, the calibration process was repeated 100 times. The mean value and standard deviation of the calibration results of the two methods are listed in Table 2 and Table 3, respectively.

It can be seen from Table 2 and Table 3 that both the IAICM and ACFCM were able to achieve an ideal calibration result when the star images were in a noiseless state. When the noise standard deviation increased to 0.2 pixels, the standard deviation of the principal point calibration error of the IAICM method was approximately 3 pixels, while the error of the ACFCM method was approximately 1 pixel, which is only 1/3 that of the IAICM method. Moreover, the focal length and the distortion calibration results were also better than those of the IAICM method, which indicates that the performance of the ACFCM proposed in this work is superior to that of the IAICM.

It should be noted that the standard deviations of the radial distortion coefficients $q_{2}$ in Table 2 and Table 3 were larger than the mean values, which was due to the corresponding distortion values from this distortion coefficient being small. Thus, it was easily disrupted by the noise and led to the corresponding calibration results oscillating around the zero value. Therefore, it was more reasonable to use the standard deviation to express the effect of this distortion term.

The ACFCM method was able to simultaneously calibrate out the mounting angles between the star sensor and gyro unit, and the calibration results are (3.0164, 29.0044, and 170.0085) degrees and (2.9205, 28.9086, and 170.0516) degrees for the different noise levels, respectively. These results obviously deteriorated after the noise increased. We proposed a calibration method for the mounting angles in another paper [24].

**Figure 5:**
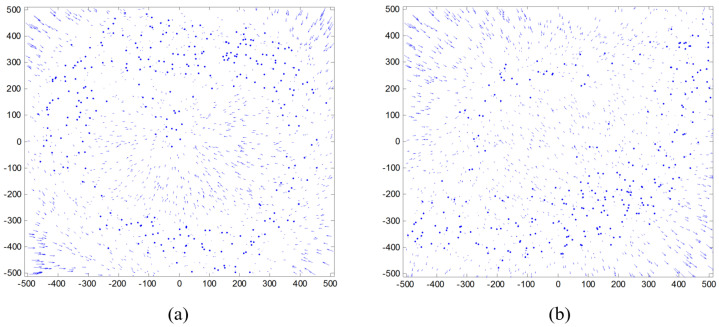
Residual error distribution after calibration for the (**a**) IAICM and (**b**) ACFCM.

Star images were projected on the image plane with the calibration results. By comparing the centroids of the projected star images and the real values, residual error vectors were obtained and their distributions are shown in Figure 5a,b. In the center of the image plane, the vector direction is in disorder and the vector length is small, indicating that the accuracy of the calibration results in these areas is relatively high. In the four corners (top-left, top-right, bottom-left, and bottom-right) of the image plane, the vector directions are more regular and the vector lengths are longer, indicating that the residuals are larger at these positions, especially in the four corners in Figure 5a. Figure 5b, on the other hand, has larger residuals in the top-left and bottom-right corners. The statistical results show that the root mean square (RMS) value of the residuals of the calibration results using the IAICM method in Figure 5a was (0.0115, 0.0115) pixels, while the RMS value of the residuals of the calibration results using the ACFCM method in Figure 5b was (0.0057, 0.0055) pixels. It is clear that the residuals using the ACFCM are only about half that of those obtained with the IAICM method, indicating that the calibration accuracy of the ACFCM method was much better.

### 4.2. Experiment

**Figure 6:**
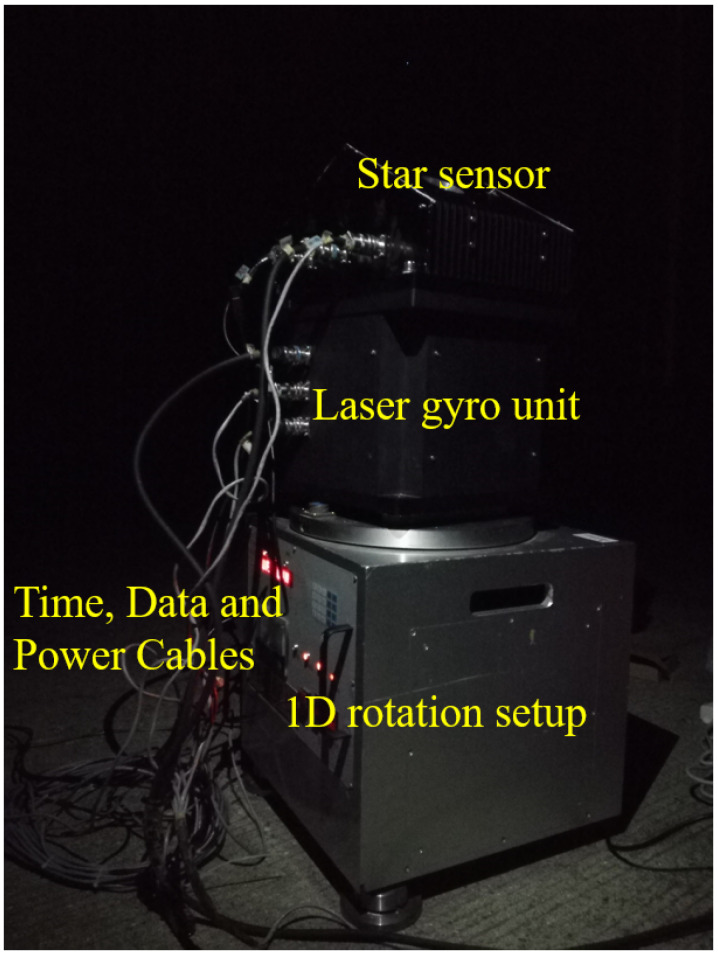
The star sensor and LGU system in the experiment.

An outdoor star observation experiment was carried out in Dawei Mountain National Forest Park (E114.1157, N28.4222, altitude 1389 m). The star sensor and a 50-type LGU experimental setup were used in the experiment, as shown in Figure 6; the 1D rotation setup was not used in the calibration experiment. The specifications of the star sensor and LGU are listed in Table 4. The start time of the calibration data acquisition for the experiment was 00:13:16 a.m. and the end time was 00:35:30 a.m. Moreover, a total of 10 groups of data were collected. Because the attitude of the star sensor changed artificially between each data group, in order to prevent the vibration instability of the star sensor before and after the attitude change, the first and last 10 frames of each data group were eliminated, and the smallest frame number in the 10 data groups was used as a benchmark to take the number of star image frames of each data group. Finally, each set contained 169 star image frames (with a time duration of less than 40 s). According to [22], the correlation time of a 50-type LGU can reach up to 500 s with an accuracy expectation of 5 arcseconds for the star sensor. Thus, the gyro errors were not considered.

**Figure 7:**
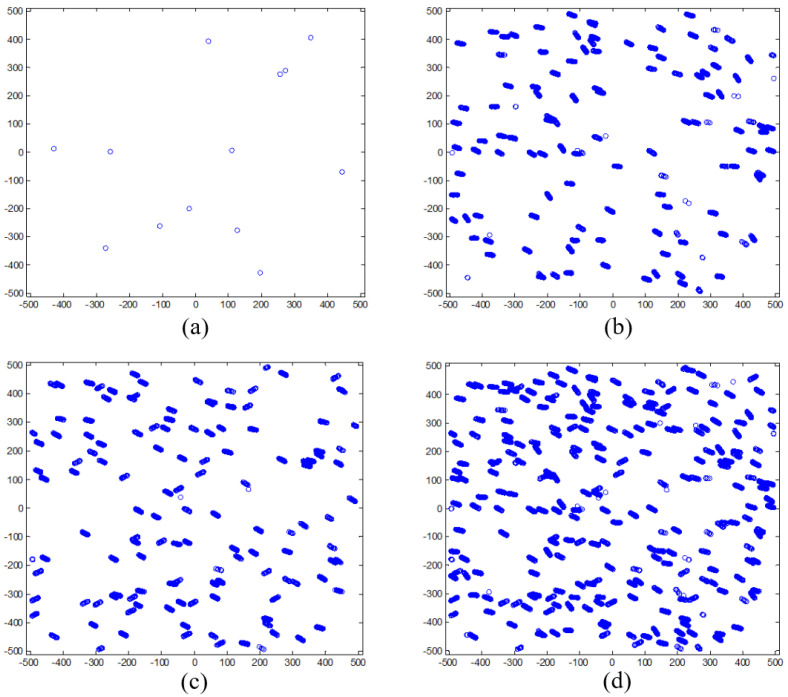
Star image distribution before and after employing the ACFCM for different groups: (**a**) star image distribution for a single-star frame; (**b**) the star image distribution for the first calibration group; (**c**) the second calibration group; and (**d**) the third calibration group.

Figure 7a is the star image distribution for a single-star image frame. It is clear that the number of star images was low and the distribution was scattered. Even though 169 star image frames were gathered, the star images in each frame were almost the same since the star sensor remained fixed for each group. The star images in the former five groups were merged into a new group named Group 1, as shown in Figure 7b,c, which show the other new group made up of the last five groups gathered. In addition, all 10 groups were merged into a third group named Group 3, as shown in Figure 7d. Compared with the single-star image frame in Figure 7a, the effective number of star images in Figure 7b–d increased significantly and the star image distribution was much more uniform, which is conducive to improving the calibration accuracy of the star sensor.

The star image in each frame was pre-processed first to reduce the noise effect, and atmospheric refraction compensation [25] was employed to reduce the atmospheric effect. As a result, the measured observation vectors were sufficiently accurate. Moreover, star images were projected by the attitude equation with the calibration results, and their centroids were compared with the real values measured using the star sensor in order to reduce the number of misrecognized stars. Taking the same star image in different frames into the calculation, the first dataset contained a total of 15,328 validly imaged stars, the second set contained 15,019 validly imaged stars, and the third set was the sum of the two sets, with 30,347 validly imaged stars. Due to the large number of identical stars (co-linear observation vectors) in each dataset, which is not conducive to the calibration of the mounting angles, the conventional attitude-dependent method was used here to calculate the internal parameters. Then, the mounting angles were calculated using the method proposed in another work [24] by us. The mounting angles were no longer considered as unknown parameters to be calibrated when applying the ACFCM method. The three sets of calibration results obtained by the IAICM and ACFCM methods are listed in Table 5 and Table 6.

From Table 5, it is easy to see that the principal point $x_{0}$ calibration results were −1.41 pixels, −10.10 pixels, and −5.21 pixels in the three groups, respectively, with a maximum difference of 8.7 pixels. In addition, the difference between the principal point $y_{0}$ calibrated from the different sets was approximately 4 pixels. Therefore, the principal point calibration results corresponding to different groups of data varied greatly and the calibration results were very unstable, which is consistent with the conclusion obtained in Section 2.4. On the other hand, the differences between the three sets of principal point calibration results obtained using the ACFCM method, as shown in Table 6, were very small, with the maximum difference between the principal point $x_{0}$ being less than 0.8 pixels, and the maximum difference between the principal point $y_{0}$ being less than 1.4 pixels. The stability of the focus calibration results of the two methods was basically the same, and their standard deviations were all approximately 0.005 mm.

Due to the large number of repeat star images, the calibration residual vectors almost completely overlapped and it was difficult to distinguish and make a judgment using the residual distribution graph. Therefore, only the root mean square (RMS) of the residuals are given here as listed in Table 7. From the table, it can be seen that the residual RMS values of the calibration results of the two methods were basically the same, with residual errors in the *x*-direction ranging from 0.1066 to 0.1207 pixels and in the *y*-direction ranging from 0.1228 to 0.1322 pixels, which resulted from the precision of the star centroids and star sensor system error. The star centroid errors directly affected the accuracy of the observation vector and should be investigated further in the future.

Undoubtedly, the Earth functions as a natural rotation table. However, the rotational velocity is relatively low at 15 arcseconds per second. This implies that the star image will not move by more than one pixel within a second when using a star sensor with an angular resolution greater than 15 arcseconds per pixel. The angle resolution of our star sensor is approximately 49 arcseconds per pixel (1024 pixels with a field of view of 14 degrees and 1024 pixels). The exposure time of the star sensor is 150 ms, indicating that the position of the star image changes by approximately 2.25 arcseconds during each exposure. Consequently, the impact of Earth’s motion (2.25/49 = 0.045 pixels) can be disregarded. The magnitude of this error caused by this is smaller than that of the star centroids. Moreover, the calibration process will require substantial time to capture an adequate number of star images, owing to the motion of the Earth. Concurrently, if the star sensor remains stationary, the star images it captures correspond to a circular ring in the sky, which is detrimental to the enhancement of calibration accuracy. For example, when the star sensor is located at the Earth’s pole, the star image will remain relatively constant. Hence, it is essential to adjust the orientation of the star sensor along three axes during the calibration procedure. As a result, the Earth’s movement is not considered in outdoor calibrations on Earth, and this has nearly become a standard practice. The motion should be theoretically considered to enhance the accuracy of the star sensor when the error of star centroids is reduced.

In summary, the main difference between the ACFCM method and the IAICM method lies in the stability of the principal point calibration. The IAICM method is insensitive to the principal point error, and the calibration results exhibited poor principal point repeatability, reliability, and accuracy, whereas the ACFCM method proposed in this work improves the principal point repeatability and reliability significantly, and produced a higher calibration result accuracy.

## 5. Conclusions

In this work, we propose an attitude-correlated frame-based calibration method (ACFCM) for a star sensor. The ACFCM method uses direct star observation, which does not require precision indoor calibration equipment. In addition, it combines the star image frames of different positions at different times, which increases the number of usable star images and improves the uniformity of the distribution of the star images. Moreover, it makes use of the strapdown gyro unit to act as a precision rotation table in order to accurately measure the attitude relationship between different star image frames. And, the gyro errors can be ignored when the correlation time is not too long. For a 50-type LGU used in the experiment, the correlation time can be as long as 500 s. The ACF method can be used to obtain high-precision attitude values, which reduces the effect of random errors on the attitude and eliminates the dependence on calibration equipment, such as a precision rotation table and star simulator. Both the simulation and experimental results verified the feasibility of the proposed ACFCM method. Compared with the IAICM method, the repeatability and reliability of the principal point obtained from the calibration of the ACFCM method proposed in this work were significantly improved, and the parameter calibration accuracy was higher. Because star sensors frequently function alongside other attitude determination systems, e.g., SINS, the installation and use of a gyro unit is no longer such a limitation.

## Figures and Tables

**Table 1:** Simulation settings for star sensor calibration.

Parameter	$x_{0}$/Pixel	$y_{0}$/Pixel	*f*/mm	$q_{1}$	$q_{2}$	$p_{1}$	$p_{2}$	$\phi_{1}$/${}^{\circ }$	$\phi_{2}$/${}^{\circ }$	$\phi_{3}$/${}^{\circ }$
True value	5	−5	25.6	$2\times 10^{-4}$	−$4\times 10^{-7}$	$2\times 10^{-4}$	$2\times 10^{-4}$	3	29	170
Initial value	0	0	25.5	0	0	0	0	0	30	160

**Table 2:** Simulation results for star sensor calibration with the IAICM.

Parameter	Noise	$x_{0}$	$y_{0}$	*f*/mm	$q_{1}$	$q_{2}$	$p_{1}$	$p_{2}$
Average value in Group 1	0.0	5.00	−5.00	25.6000	$2\times 10^{-4}$	−$4\times 10^{-7}$	$2\times 10^{-4}$	$2\times 10^{-4}$
Std value in Group 1	0.0	0.00	0.00	0.0000	0.00	0.00	0.00	0.00
Average value in Group 2	0.0	4.89	−5.02	25.5999	$2.01\times 10^{-4}$	-$5.38\times 10^{-7}$	$1.99\times 10^{-4}$	$2\times 10^{-4}$
Std value in Group 2	0.1	1.23	1.21	0.0013	$1.84\times 10^{-5}$	$1.9\times 10^{-6}$	$6.55\times 10^{-6}$	$6.2\times 10^{-6}$
Average value in Group 3	0.0	4.73	−5.49	25.6002	$1.96\times 10^{-4}$	$5.16\times 10^{-8}$	$1.98\times 10^{-4}$	$1.97\times 10^{-4}$
Std value in Group 3	0.2	3.02	2.79	0.0026	$3\times 10^{-5}$	$3.77\times 10^{-6}$	$1.56\times 10^{-5}$	$1.43\times 10^{-5}$

**Table 3:** Simulation results for star sensor calibration with the ACFCM.

Parameter	Noise	$x_{0}$	$y_{0}$	*f*/mm	$q_{1}$	$q_{2}$	$p_{1}$	$p_{2}$
Average value in Group 1	0.0	5.00	−5.00	25.6000	$2\times 10^{-4}$	−$4\times 10^{-7}$	$2\times 10^{-4}$	$2\times 10^{-4}$
Std value in Group 1	0.0	0.00	0.00	0.0000	0.00	0.00	0.00	0.00
Average value in Group 2	0.0	5.05	−4.97	25.6000	$1.96\times 10^{-4}$	$1.55\times 10^{-7}$	$1.99\times 10^{-4}$	$2.02\times 10^{-4}$
Std value in Group 2	0.1	0.45	0.56	0.0007	$1.0\times 10^{-6}$	$1.88\times 10^{-6}$	$1.65\times 10^{-4}$	$1.66\times 10^{-4}$
Average value in Group 3	0.0	5.03	−4.92	25.6002	$1.97\times 10^{-4}$	−$5.82\times 10^{-8}$	$2.02\times 10^{-4}$	$1.98\times 10^{-4}$
Std value in Group 3	0.2	0.99	1.12	0.0014	$2.15\times 10^{-5}$	$2.29\times 10^{-6}$	$5.49\times 10^{-6}$	$5.55\times 10^{-6}$

**Table 4:** Specifications of the star sensor and LGU used in the experiment.

Parameters	Values	Parameters	Values
Resolution	1024 × 1024	Focus length	25.6 mm
FOV	$14^{\circ }\times 14^{\circ }$	Exposure time	150 ms
Star sensor update frequency	5 Hz	Gyro sampling frequency	100 Hz
Gyro bias	0.01${}^{\circ }$/h	Random walk noise	0.003${}^{\circ }/\sqrt{h}$

**Table 5:** Experiment results for the star sensor calibration with the IAICM.

Parameter	$x_{0}$	$y_{0}$	*f*/mm	$q_{1}$	$q_{2}$	$p_{1}$	$p_{2}$
Group 1	−1.41	13.20	25.6555	−$4.72\times 10^{-4}$	$8.31\times 10^{-6}$	−$2.14\times 10^{-7}$	$6.34\times 10^{-6}$
Group 2	−10.10	17.13	25.6453	−$3.24\times 10^{-4}$	−$9.18\times 10^{-6}$	−$8.68\times 10^{-6}$	$8.67\times 10^{-6}$
Group 3	−5.21	14.56	25.6495	−$3.9\times 10^{-4}$	−$8.58\times 10^{-6}$	−$3.3\times 10^{-6}$	$6.58\times 10^{-6}$

**Table 6:** Experiment results for the star sensor calibration with the ACFCM.

Parameter	$x_{0}$	$y_{0}$	*f*/mm	$q_{1}$	$q_{2}$	$p_{1}$	$p_{2}$
Group 1	−8.26	15.52	25.6582	−$4.98\times 10^{-4}$	$1.05\times 10^{-5}$	−$1.0\times 10^{-5}$	$1.1\times 10^{-5}$
Group 2	−7.75	16.86	25.6464	−$3.35\times 10^{-4}$	$8.56\times 10^{-6}$	−$4.72\times 10^{-6}$	$1.02\times 10^{-5}$
Group 3	−7.50	15.77	25.6519	−$4.14\times 10^{-4}$	$1.07\times 10^{-6}$	−$6.31\times 10^{-6}$	$9.91\times 10^{-6}$

**Table 7:** Residual error of the ACFCM and IAICM.

Group	No. 1 $\left(x_{0},y_{0}\right)$	No. 2 $\left(x_{0},y_{0}\right)$	No. 3 $\left(x_{0},y_{0}\right)$
IAICM	(0.1066, 0.1241)	(0.1206, 0.1319)	(0.1131, 0.1294)
ACFCM	(0.1082, 0.1228)	(0.1207, 0.1322)	(0.1142, 0.1287)

## Data Availability

Data are contained within the article.
